# Supplementary material for: In Situ Raman Analysis of Biofilm Exopolysaccharides Formed in Streptococcus mutans and Streptococcus sanguinis Commensal Cultures
Source: Int J Mol Sci. 2023 Apr 3;24(7):6694. doi: 10.3390/ijms24076694 (PMC10095091; doi:10.3390/ijms24076694)
Supplement: Supplementary file 1 [file ijms-24-06694-s001.zip › ijms-2257961-supplementary.pdf]

Supplementary Information

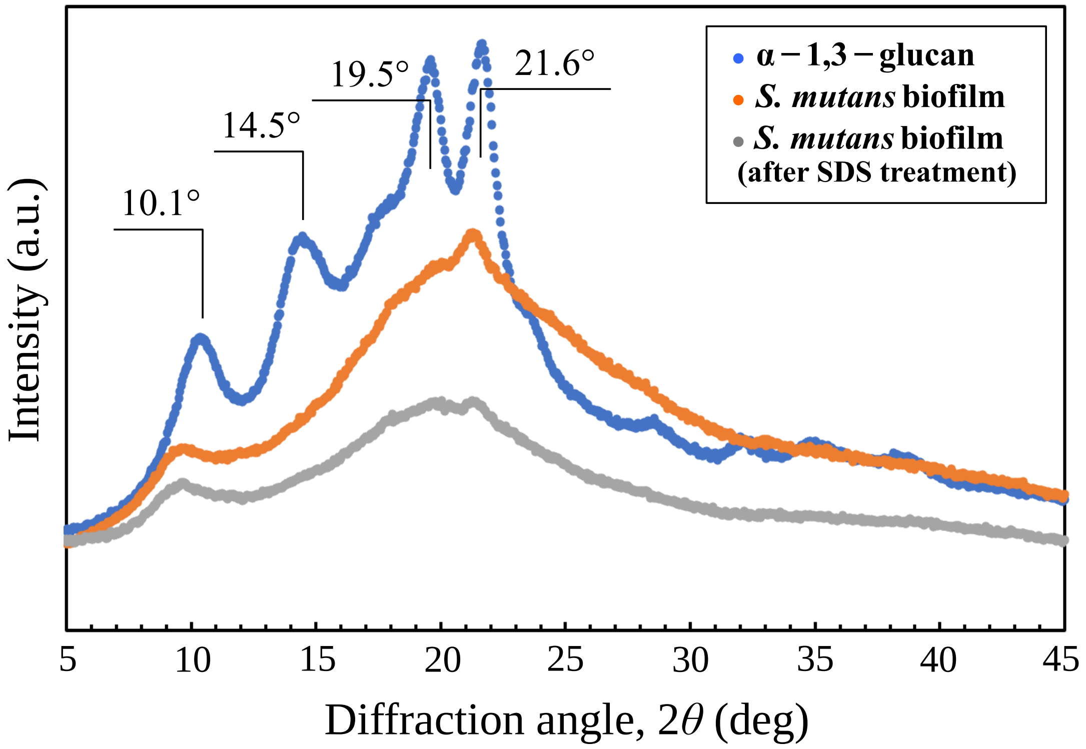

Figure S1: Comparison between Wide-Angle X-ray Diffraction (WAXD) of synthetic α − 1,3 – glucans and *S. mutans* biofilm before and after Smith degradation. The peaks at the diffraction angles in inset correspond to α − 1,3 – glucans. WAXD data revealed a lower crystallinity for biofilm α − 1,3 – glucans as compared to the synthetic α − 1,3 – glucan sample,[1] although the crystalline fraction appeared to survive SDS treatment.

WAXD spectra were obtained using a MicroMax-007HF system (Rigaku Co., Tokyo, Japan) operated at 40 kV and 30 mA with Cu Kα radiation (λ = 0.15418 nm) in transmission geometry mode and in a high vacuum. The X-ray diffraction pattern was recorded on an imaging plate (BAS-SR 127, 2540 × 2540 pixels, 50 × 50 mm^2/pixel; Fujifilm Co., Tokyo, Japan) and read with a RAXIA-D*i* system (Rigaku Co., Tokyo, Japan). The sample-to-detector distance was set at approximately 83 mm. The sample holder was maintained at room temperature, and the measurements were all performed at room temperature. WAXD pattern analysis was performed using 2DP software (Rigaku Co. Tokyo, Japan).

[1]Y. Hori, Y. Enomoto, S. Kimura, T. Iwata, Synthesis of α − 1,3 – and β-1,6-glucan esters with carbon-carbon double bonds and their surface modification, *Polym. Int.* **70,** 573-581 (2021).

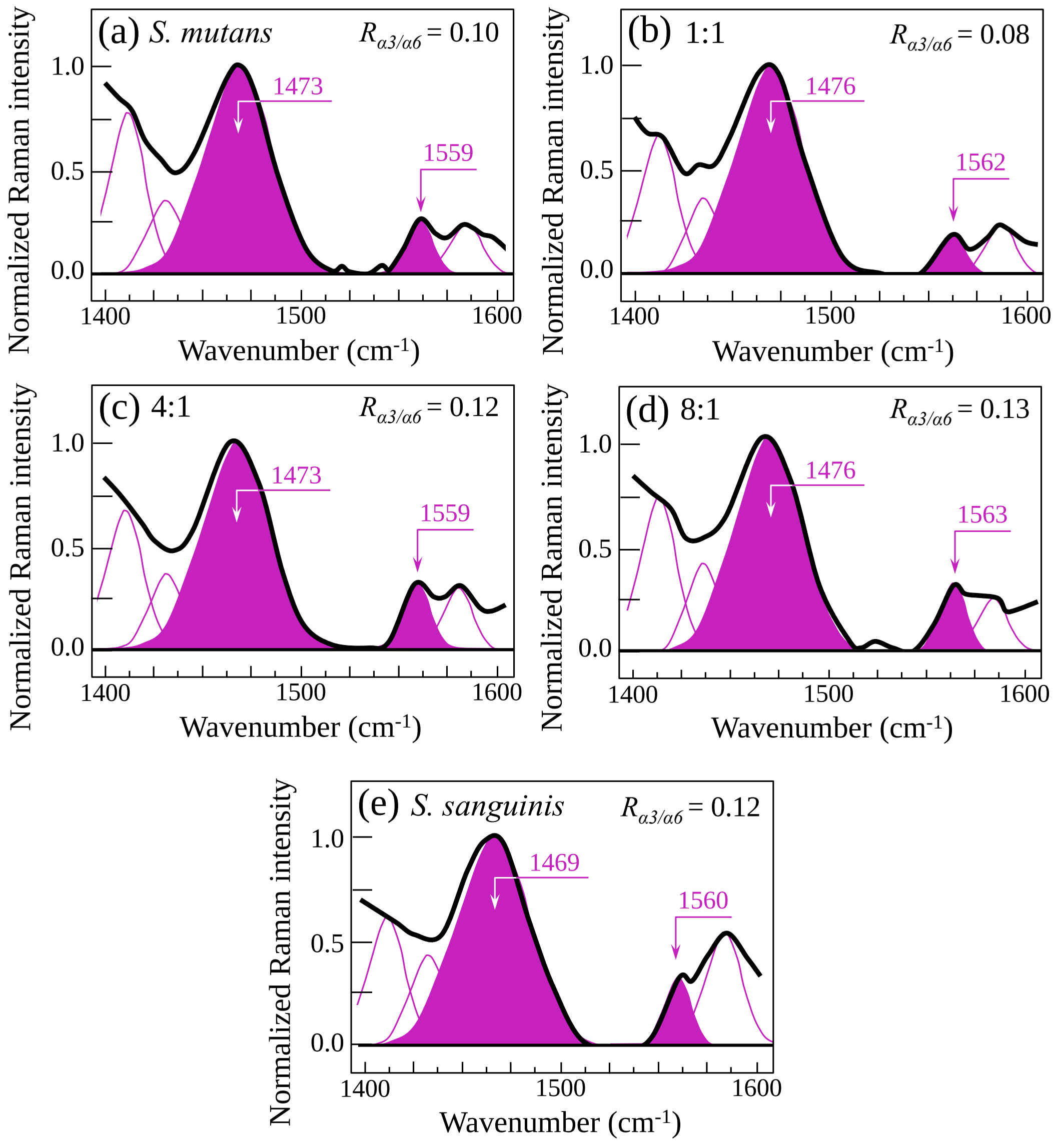

Figure S2: Raman spectra in the spectral zone 1400~1600 cm^{-1}, as collected on cultures/cocultures (at 24 h) with *S. mutans* to *S. sanguinis* ratios of: (a) 1:0, (b) 1:1, (c) 4:1, (d) 8:1, and (e) 0:1; the band at ~1484 cm^{-1} is common to both α – 1,3 – to α – 1,6 – glucan structures (H – C6 – OH bending), while the band at 1568 cm^{-1}, only seen in the spectrum of α – 1,3 – glucan, relates to the presence of C=C bonds. Values of the Raman intensity ratio, $R^{(3)}_{\alpha3/\alpha6} = I_{1568}/I_{1482}$, are reported in inset to each figure. The 1568 cm^{-1} signal from C=C bonds is a cumulative one arising from the glucansucrase enzyme used by *S. mutans* to synthesize the α – 1,3 – glucan structure, to tryptophan in cellular proteins, and to the formation of transient ring structures during oxidation of α – 1,3 – glucans by oxygen radicals produced by *S. sanguinis*. This point is discussed in details in the main text.

Wavenumber (cm^{-1})	Vibrational origin	References
478	S−S stretching (ring structure)	[46]
493	C−C−C bending (in glycogen)	[45,47,48]
520	C2−C3−OH deformation (only in α−1,6−glucan)	[45]
531	C2−C3−O(−C1) deformation (only in α−1,3−glucan)	[45]
555	C2−C1−O deformation (in both α−1,3− and α−1,6−glucan)	[45]
572	C5−C6−O(−H) deformation (only in α−1,3−glucan)	[45]
919	C1−O−C6 stretching (in both α−1,3− and α−1,6−glucan)	[45]
948	C1−O−C3 stretching (in both α−1,3− and α−1,6−glucan)	[43-45]
1484	H−C6−H bending (in both α−1,3− and α−1,6−glucan)	[45]
1568	C=C stretching (extrinsic to glucan structure)	[31]
1724<br>1747<br>1768<br>1788	C=O stretching (carbonyl units)	[52]

Table S1: Wavenumbers, vibrational origins, and literature references for the main Raman bands discussed in the main text and figures. The reference numbers refer to the reference list given in the text.
